# Trends in food insecurity rates at an academic primary care clinic: a retrospective cohort study

**DOI:** 10.1186/s12887-021-02829-3

**Published:** 2021-08-27

**Authors:** Kimberly Montez, Callie L. Brown, Arvin Garg, Scott D. Rhodes, Eunyoung Y. Song, Alysha J. Taxter, Joseph A. Skelton, Laurie W. Albertini, Deepak Palakshappa

**Affiliations:** 1 Department of Pediatrics, Wake Forest University Health Sciences, Winston-Salem, NC 27157 USA; 2 Departments of Pediatrics and Epidemiology and Prevention, Wake Forest University Health Sciences, Winston-Salem, NC 27157 USA; 3 Department of Pediatrics, University of Massachusetts Medical School, Worcester, MA 01655 USA; 4 Department of Social Sciences and Health Policy, Wake Forest University Health Sciences, Winston-Salem, NC 27101 USA; 5 Health Quality Partners, Doyleston, PA USA; 6 Departments of Pediatrics and Epidemiology and Prevention, Wake Forest University Health Sciences, Winston-Salem, NC 27101 USA; 7 Departments of Pediatrics and Internal Medicine, Wake Forest University Health Sciences, Winston-Salem, NC 27157 USA

**Keywords:** Food insecurity, Social determinants of health, Primary care

## Abstract

**Background:**

Healthcare organizations are increasingly screening and addressing food insecurity (FI); yet, limited data exists from clinic-based settings on how FI rates change over time. The objective of this study was to evaluate household FI trends over a two-year period at a clinic that implemented a FI screening and referral program.

**Methods:**

In this retrospective cohort study, data were extracted for all visits at one academic primary care clinic for all children aged 0–18 years whose parents/guardians had been screened for FI at least once between February 1, 2018 to February 28, 2019 (Year 1) and screened at least once between March 1, 2019 to February 28, 2020 (Year 2). Bivariate analyses tested for differences in FI and demographics using chi-square tests. Mixed effects logistic regression was used to assess change in FI between Years 1 and 2 with random intercept for participants controlling for covariates. The interaction between year and all covariates was evaluated to determine differences in FI change by demographics.

**Results:**

Of 6182 patients seen in Year 1, 3691 (59.7%) were seen at least once in Year 2 and included in this study. In Year 1, 19.6% of participants reported household FI, compared to 14.1% in Year 2. Of those with FI in Year 1, 40% had FI in Year 2. Of those with food security in Year 1, 92.3% continued with food security in Year 2. Compared to Hispanic/Latinx participants, African American/Black (OR: 3.53, 95% CI: 2.33, 5.34; *p* < 0.001) and White (OR: 1.88, 95% CI: 1.06, 3.36; *p* = 0.03) participants had higher odds of reporting FI. African American/Black participants had the largest decrease in FI between Years 1 and 2 (− 7.9, 95% CI: − 11.7, − 4.1%; *p* < 0.0001).

**Conclusions:**

Because FI is transitional, particularly for racial/ethnic minorities, screening repeatedly can identify families situationally experiencing FI.

## Background

Food insecurity (FI), the lack of dependable access to sufficient affordable, nutritious foods for an active and healthy life [1], is a public health dilemma in the United States (US). In 2019, an estimated 35.2 million people in the US had FI (10.5%); households with children disproportionately experienced FI (13.6%), affecting 10.7 million children nationwide [1]. FI is known to negatively impact child physical, developmental, and mental health outcomes [2, 3]. Due to the prevalence and association with negative child health outcomes, both the American Academy of Pediatrics (AAP) recommends that providers routinely screen families for household FI [4, 5]. Health care institutions, payers, and stakeholders are increasingly recognizing the importance of screening patients for and addressing FI in clinical settings [6–8].

While there is a growing body of research focused on the process of screening for FI in healthcare settings and referring families for resources, there are still limited data from clinic-based settings on changes in FI rates over time [9, 10]. FI is often transient, with children and families moving in and out of FI over time due to changes in family circumstances or seasonal fluctuations in food availability due, for example, to changes in household expenses, and availability of school meals [11–13]. However, to date, the majority of studies evaluating programs that screen and address FI in clinical care settings have been cross-sectional [3, 14, 15]. Few studies have demonstrated FI patterns longitudinally among a cohort of pediatric participants in a clinical setting [16].

The objective of this study was to evaluate the trends in FI rates over a two-year period among a cohort of pediatric participants at one urban, academic primary care clinic that had implemented a FI screening and referral program.

## Methods

### Study overview and participants

We conducted a retrospective cohort study of children ages 0–18 years old within families that were screened for FI between February 1, 2018 and February 28, 2020 at an academic pediatric primary care clinic. This clinic receives approximately 19,000 visits annually, including 11,000 well child visits and 8000 acute care visits, and serves a primarily low-income, urban, Medicaid-insured population. The clinic is located in medium-sized city within Forsyth County, North Carolina and serves a community in which 22.7% of the population is under 18 years; 27.5% report being African American/Black, 13.3% Hispanic/Latinx, and 56.3% non-Hispanic White; 34% of persons aged 25 years and older have a Bachelor’s degree or higher; and 15.2% live in poverty [17]. The clinic is the teaching site for the pediatric residency program, including continuity clinics. The clinic is staffed by 15 attending physicians who primarily supervise the residents, 5 of whom directly provide patient care. There are 38 residents who provide care; 16 are first year residents. All providers receive an orientation regarding clinic procedures, including FI screening and referral, as well as periodic FI lectures throughout the year [9].

Beginning in January 2018, the clinic began systematically screening all patients and families with a child presenting for any visit type (including well-child, return, and urgent visits) for household FI using a paper-based form; prior to that, the clinic had been screening verbally since 2014 [9]. The paper-based questionnaire screened patients for several unmet social needs, including housing instability, lack of transportation, utility insecurity, intimate partner violence, and legal problems, and it included the Hunger Vital Sign™ (HVS) to screen families for household FI. The HVS was developed for use as a clinical screening measure and has been recommended by the AAP for FI screening at pediatric health supervision visits [4, 18]. As the pediatric patient was accompanied to an exam room, the nurse provided the parent or guardian with the paper-based questionnaire in English or Spanish depending on the language preference of the parent or guardian. Parents/guardians of children and adolescents completed the questionnaire prior to being seen by the clinician. The results of the written questionnaire were then reviewed by the clinician at the time of the visit, discussed with the family, and entered into the visit documentation template in discreet data fields. The documentation rate for providers was approximately 97% [9]. Resources available at the time of the visit included (1): a bag of non-perishable food items to feed a family of four for 3–4 meals (2), a list of local community-based hunger relief resources (e.g. food pantries, mobile food programs, etc.) (3), meeting with the clinic’s on-site care coordinator to discuss and assist families with obtaining additional community resources (e.g. cooking programs) if the family was interested, and (4) for those meeting with the care coordinator, a follow up phone call, and (5) referrals to federal nutrition programs (e.g. Supplemental Nutrition Assistance Program).

We extracted data from the electronic health record (EHR) for all patients who had been screened for FI at least once between February 1, 2018 to February 28, 2019 (Year 1) and screened at least once between March 1, 2019 to February 28, 2020 (Year 2). Patients were excluded if they were not 0–18 years of age during Year 1 for a total study population of 3691 children.

### Food insecurity

The HVS screening measure is used by our clinic to screen for household FI at every visit and therefore served as the basis for assessing FI status in this study. The HVS includes two questions: “Within the past 12 months we worried whether our food would run out before we got money to buy more” and “Within the past 12 months, the food we bought just didn’t last and we didn’t have the money to get more.” [18] The validated HVS by Hager et al. uses the answer responses of “often true,” “sometimes true,” “never true,” and “don’t know/refused.” Our clinic modified the HVS answer choices to binary response options, “yes” or “no.” A response of “yes” by the caregiver to either question indicates a positive screen for household FI. Because the HVS asks about FI within the 12 months, a family was considered to have FI at least once during Year 1 or FI at least once during Year 2 if they screened positive for FI at any visit during that year. Our outcome of interest was the change in FI over time between Year 1 and Year 2.

### Patient demographics

We extracted demographic data from the EHR for each participant screened, including age (categorized in this analysis as 0 to < 6 years of age; 6 to < 12 years of age, > = 12 years of age), sex, race, and ethnicity. Race/ethnicity was categorized for this analysis as self-identified non-Hispanic White, non-Hispanic African American/Black, Hispanic/Latinx, or other; the other category included self-identification as more than one race. We also extracted the preferred language reported by the caregiver (English, Spanish, or other) and insurance type. Insurance type was categorized as public, private, or self-pay. We also assessed if the caregiver of a participant received any type of resource (e.g. food bag, care coordinator meeting) at any visit during Year 1.

### Statistical analyses

Participant characteristics were presented as N (percent) for categorical variables. Bivariate analyses tested for differences in patient demographics between participants who were included and participants who were not using chi-square tests. In primary analysis, mixed effects logistic regression was used to assess the change in FI between Year 1 and Year 2 with random intercept for the individual participant and controlling for age, sex, race/ethnicity, language, and insurance. Because changes in national FI rates have varied based on demographic characteristics, a priori it was hypothesized that there may be differences in the change in FI over time based on the demographic characteristics of participants (age group, sex, race/ethnicity, language, and insurance status) [1, 19]. In order to evaluate these potential differences, the interaction between the year and all covariates was evaluated to determine if differences in the change in FI varied by participant demographics in secondary analyses. A potential interaction (*p* < 0.2) between year and race/ethnicity was found, and the predicted probability of FI between groups over time was estimated using the *margins* command in Stata. Two-sided hypothesis test was used, and an alpha < 0.05 was considered significant. All statistical analyses were conducted using Stata 15.0 (StataCorp, College Station, TX). The Wake Forest School of Medicine Institutional Review Board approved this study for expedited review (#IRB00062179). This research met the criteria for a waiver of consent entirely according to 45 CFR 46(d).

## Results

### Study population characteristics

Of the 6182 patients seen in Year 1, 3691 (59.7%) were seen at least once in Year 2 and included in this study (Table 1). The 2491 (40.3%) patients who were not included were significantly more likely to be older age, but there were no significant differences in FI rates or other covariates at baseline. Of the 3691 participants included in this analysis, the majority of their caregivers reported being Hispanic/Latinx (63.8%), having Medicaid or other public insurance (94%), and speaking Spanish (55.2%). About half of participants were less than 6 years of age, 31% were 6–12 years, and 20% were 12–18 years old. In Year 1, 19.6% of caregivers reported FI to their pediatric provider, compared to 14.1% in Year 2.

**Table 1:** Study population baseline demographics

		Total population: N (%)
		3691
**Sex**	Female	1783 (48.3)
	Male	1908 (51.7)
**Age group**	0 to < 6 years of age	1790 (48.5)
	6 to < 12 years of age	1145 (31.0)
	12 to < 19 years of age	756 (20.5)
**Language**	English	1600 (43.4)
	Spanish	2036 (55.2)
	Other	55 (1.5)
**Race/Ethnicity**	Hispanic/Latinx	2353 (63.8)
	Af. Am./Black	770 (20.9)
	White	190 (5.2)
	Other	378 (10.2)
**Insurance**	Medicaid	3467 (94.0)
	Private	75 (2.0)
	Self-pay	149 (4.0)
**Food insecurity year 1**	Yes	723 (19.6)
**Food insecurity year 2**	Yes	521 (14.1)

*Af. Am.* African American

### Change in food insecurity over time

**Fig. 1:**
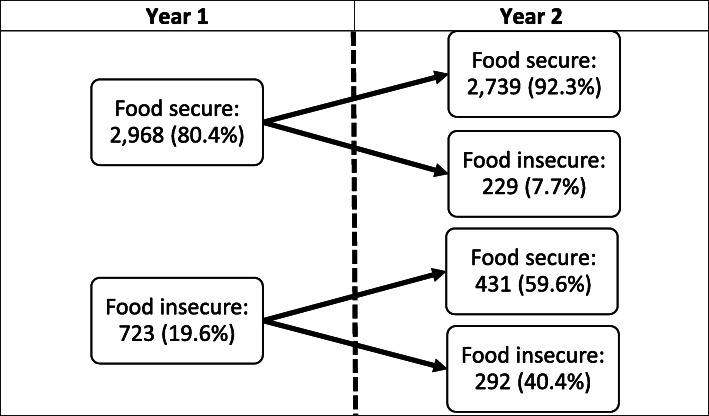
Change in food insecurity responses over time

The change in FI is demonstrated in Fig. 1. Of those who reported food security in Year 1, most (92.3%) continued to report food security in Year 2. Of the 723 participants’ caregivers who identified as having FI in Year 1, 657 (90.9%) had documentation in the EHR indicating that they had received at least one resource in the clinic at one of their visits during Year 1. Of those who screened positive for FI in Year 1, less than half (40%) continued to screen positive for FI in Year 2. Caregivers of participants who continued to screen positive for FI in Year 2 were significantly more likely to have received at least one resource in Year 1 than caregivers of participants who had screened positive for FI in Year 1, but then screened negative in Year 2 (93.8% vs 88.9%, *p* = 0.02). Among all cohort participants, the majority (75.2%) of caregivers remained food secure between Year 1 and Year 2, while 6.2% transitioned from having food security to having FI, and 11.7% transitioned in the opposite direction.

**Fig. 2:**
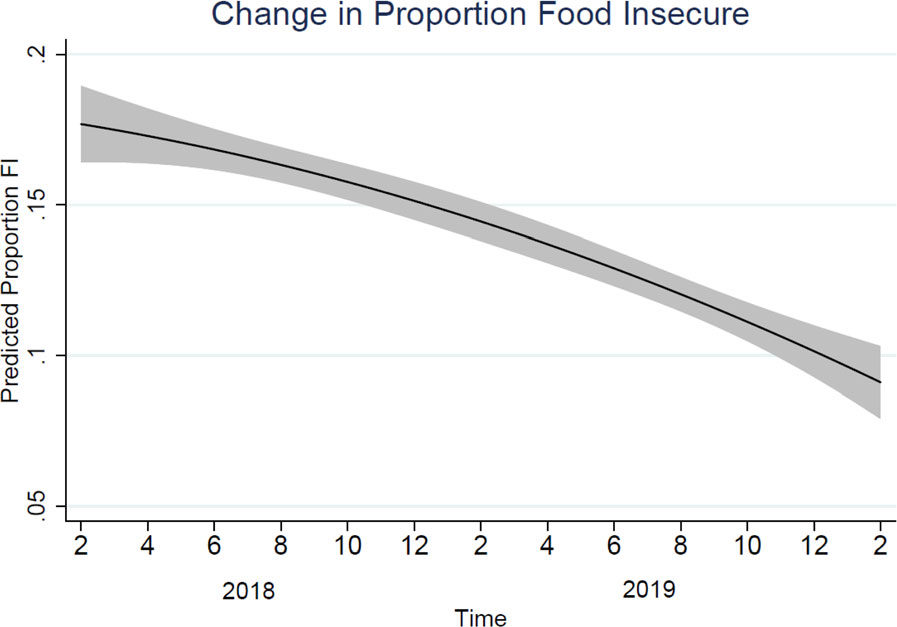
Change in the predicted proportion of participants reporting food insecurity per month

Between both years, 7.9% continued to report FI. Between Year 1 and Year 2, we found a decline in the predicted proportion of caregivers who screened positive for FI (Fig. 2).

In mixed effects models controlling for age, sex, race/ethnicity, language, and insurance status, participants’ caregivers had a significantly lower odds of reporting FI in Year 2 compared to Year 1 (OR: 0.56, 95% CI: 0.48, 0.66; *p* < 0.001) (Table 2). Among the study population, families with children who were 6–12 years of age (OR: 0.74, 95% CI: 0.57, 0.96; *p* = 0.02) and greater than 12 years of age (OR: 0.48, 95% CI: 0.36, 0.65; *p* < 0.001) had a lower odds of reporting FI during the study period compared to families with children between 0 and 6 years of age. Compared to the Hispanic/Latinx group, African American/Black (OR: 3.53, 95% CI: 2.33, 5.34; *p* < 0.001), White (OR: 1.88, 95% CI: 1.06, 3.36; *p* = 0.03), and other racial groups (OR: 2.90, 95% CI: 1.83, 4.60; *p* < 0.001) had a higher odds of reporting FI.

**Table 2:** Multivariable model evaluating odds of reporting food insecurity during the study period

		OR (95% CI)	***p***-value
**Year**	1	Ref	
	2	0.56 (0.48, 0.66)	**< 0.001**
**Sex**	Female	Ref	
	Male	0.91 (0.73, 1.14)	0.43
**Age group**	0 to < 6 years of age	Ref	
	6 to < 12 years of age	0.74 (0.57, 0.96)	**0.02**
	12 to < 19 years of age	0.48 (0.36, 0.65)	**< 0.001**
**Language**	English	Ref	
	Spanish	1.12 (0.78, 1.61)	0.55
	Other	0.61 (0.23, 1.60)	0.31
**Race/Ethnicity**	Hispanic/Latinx	Ref	
	Af. Am./Black	3.53 (2.33, 5.34)	**< 0.001**
	White	1.88 (1.06, 3.36)	**0.03**
	Other	2.90 (1.83, 4.60)	**< 0.001**
**Insurance**	Public	Ref	
	Private	0.22 (0.09, 0.54)	**0.001**
	Self-pay	0.92 (0.55, 1.51)	0.73

*Af. Am.* African American

### Change in food insecurity over time by demographics

In secondary analyses, a potential interaction between race/ethnicity and year was identified, and the predicted probability of FI between groups was determined over time while controlling for all other covariates. We found a significant decrease in the percent of participants’ caregivers who reported FI among all racial/ethnic groups (Table 3). African American/Black caregivers were found to have the largest decrease in self-reported FI between Year 1 and Year 2 (− 7.9, 95% CI: − 11.7, − 4.1%; *p* < 0.0001). Caregivers who reported being Hispanic/Latinx were found to have the smallest decrease in self-reported FI between Year 1 and Year 2 (− 1.8, 95% CI: − 2.7, − 0.9%; *p* = 0.0001). We did not find a potential interaction between year and all other covariates, including age group, sex, language, and insurance status.

**Table 3:** Change in predicted proportion of food insecurity by race/ethnicity

	Year 1: Predicted % (95% CI)	Year 2: Predicted % (95% CI)	Adjusted percent difference: % (95% CI)	***p***-value
**Hispanic/Latinx**	5.2 (3.8, 6.5)	3.4 (2.4, 4.3)	−1.8 (− 2.7, − 0.9)	0.0001
**African American/Black**	17.5 (12.6, 22.5)	9.6 (6.4, 12.9)	−7.9 (− 11.7, − 4.1)	< 0.0001
**White**	12.0 (5.7, 18.3)	4.2 (1.4, 7.0)	− 7.8 (− 13.5, − 2.2)	0.007
**Other**	15.0 (9.5, 20.5)	8.0 (4.5, 11.5)	− 7.0 (− 11.7, − 2.3)	0.004

All analyses were controlling for covariates including age, gender, race/ethnicity, insurance status, and language

## Discussion

Over a two-year period, a statistically significant decline in the proportion of participants who screened positive for FI was demonstrated at one academic pediatric primary care clinic serving primarily Medicaid-insured patients that had implemented a FI screening and referral program. Of those who reported FI in Year 1, less than half continued to report FI in Year 2; the vast majority of participants’ caregivers who reported having food security in Year 1 continued to report food security in Year 2. There was a significant decrease in self-reported FI between the 2 years among all races/ethnicities, although African American/Black participants had the largest decrease, while Hispanic/Latinx participants were found to have the smallest decrease.

There is growing interest among healthcare organizations in addressing FI in clinical settings [4–8]; yet, to date most studies have been cross-sectional [3, 14, 15]. Our results add to the existing literature by examining the longitudinal trends of FI rates among a large cohort of low-income, racially/ethnically diverse pediatric participants at one academic primary care clinic that had implemented a FI screening and referral program. Overall, prior to the Coronavirus Disease 2019 pandemic (COVID-19), we found a decrease in FI rates between 2 years of a clinic-based FI screening and referral program, with a majority of participants reporting FI transitioning from having FI to having food security (59.6%). While it remains to be seen how trends will change with COVID-19, FI rates are likely to increase, with nationally representative surveys demonstrating record rates among respondents with children [20, 21]. This study’s findings are similar to another longitudinal study in a non-clinical setting in which children among a kindergarten cohort undergoing FI screening at two assessments were found to have food security at both assessments (80%), transition to FI (6%), and FI at both assessments (7%); however, in our study, more participants transitioned from having FI to reporting food security, possibly associated with clinic-based FI interventions [13]. Authors of a previous clinic-based longitudinal study found smaller percentages of transitions in FI status and FI persistence among a cohort of pediatric participants aged 0–3 years who were screened for FI multiple times and provided clinic-based interventions [16]. The majority of our cohort included households with children 6 years of age and older, which are known to be at lower risk of having FI compared to children younger than 6 years of age [1], making transitions to having food security more likely for our older cohort.

There are several potential reasons why we may have seen in a decline in FI rates among the study population. The first possible explanation for this study’s finding of a decreased trend in FI rates is that the clinic-based screening and referral program was effective at mitigating FI. In bivariate analysis though, participants who continued to screen positive for FI in Year 2 were significantly more likely to have received at least one resource in Year 1 than participants who had screened positive for FI in Year 1, but then screened negative in Year 2. However, this difference in receiving resources was small. While it is possible that an unaccounted for outside factors led to the significant decreased trend in FI rates among the study population, we are not aware of any major changes that occurred to the local food system or availability of resources during the time period studied. For example, locally in Forsyth County, FI rates in households with children had also been declining between 2017 (17%) and 2018 (15.2%), the most recent year for which local FI data is available [22]. Second, the observed decrease in FI rates is consistent with local and national data prior to the COVID-19 pandemic, which have similarly shown a decrease in FI in recent years. In the US, FI peaked in 2009 with 21.3% of households with children reporting FI, and in 2018 and 2019 these rates continued to decrease to 13.9 and 13.6%, respectively [1, 23]. Third, during the second year, participants would have been 1 year older, making FI risk lower since households with older children are at lower FI risk [1]. A fourth possibility is that participants stopped admitting to FI on the paper-based survey, either due to survey fatigue, lack of confidence in clinic-based interventions, stigma, or fear of public charge [24–29]. A fifth possible explanation is that, because of the transient nature of FI, the screening occurred during a season or family circumstance in which the family had food security, although the HVS assesses FI over the previous 12-month period, which would have accounted for all four seasons.

Interestingly, we found that changes in FI rates over time varied by race/ethnicity. African American/Black participants were found to have the largest decrease in self-reported FI between Years 1 and 2, followed by White patients. Hispanic/Latinx patients were found to have the smallest decrease. National data in recent years prior to the COVID-19 pandemic also demonstrated a significant decrease in FI rates among African American/Black households, while rates in Hispanic/Latinx households with children remained relatively unchanged [1]. One possible explanation for this study’s finding is that Hispanic/Latinx households may be less likely to attend medical visits or to admit to FI on surveys, possibly due to uncertainty in the context of “public charge” and/or fear of deportation, especially if there is mixed immigration status among family members [30, 31]. Studies have shown that children living in immigrant families are less likely to access health care resources or public benefits, even if the qualifying child is a US citizen [28, 32–35].

Despite the strengths of the study, there are several limitations that should be acknowledged. First, because this study was conducted at a single primary care clinic that serves predominantly low-income, Medicaid-insured, and racial/ethnic minority patients, the results may not be generalizable to other clinical settings or pediatric populations. Second, because the self-report of FI status by parents utilized a dichotomous response for the Hunger Vital Sign™ rather than a Likert scale (never, sometimes true, or often true), which are more sensitive, families with FI may have been missed [36]. Third, because the FI disclosure rates were reliant on results that were documented in the EHR, we cannot be sure that clinicians accurately documented the results in the EHR. However, in our prior study, we found that almost 97% of providers documented the results of the FI screen after the implementation of our paper-based screening [9]. Fourth, we were unable to examine key determinants of FI due to the limitations of currently available data within the EHR, such as household income, parental age, household size or composition, and participation in nutritional assistance programs. Fifth, because of the limitations of the data available in the EHR, we are unable to determine which particular resources or how many resources families that screened positive for FI received. We were only able to evaluate if patients received any resources. Seventh, we were unable to determine causality of the program since there was no control group.

## Conclusions

Our study contributes to the understanding of longitudinal changes in FI status among a cohort of pediatric participants in a clinical setting following implementation of a FI screening and referral program. Our findings add to the current body of knowledge that FI is often transitional, reinforcing the importance of longitudinal screening for household FI in the primary care setting, particularly for racial/ethnic minorities. Screening repeatedly can identify families that situationally experience household FI. Further research is needed to determine which interventions are most effective at mitigating FI in a primary care setting.

## Data Availability

The data that support the findings of this study are not openly available due to them containing information that could compromise research participant privacy/consent and a data sharing mandate; data are available from the corresponding author [KM] upon reasonable request.

## Abbreviations

AAP: Academy of Pediatrics
COVID-19: Coronavirus Disease 2019
EHR: electronic health record
FI: food insecurity
HVS: Hunger Vital Sign™
US: United States
